# A Sandwich Structural Filter Paper–AgNWs/MXene Composite for Superior Electromagnetic Interference Shielding

**DOI:** 10.3390/polym16060760

**Published:** 2024-03-10

**Authors:** Xiaoshuai Han, Hongyu Feng, Wei Tian, Kai Zhang, Lei Zhang, Jiangbo Wang, Shaohua Jiang

**Affiliations:** 1 State Key Laboratory of Biobased Material and Green Papermaking, Qilu University of Technology, Shandong Academy of Sciences, Jinan 250353, China; xiaoshuai.han@njfu.edu.cn (X.H.);; 2 Jiangsu Co-Innovation Center of Efficient Processing and Utilization of Forest Resources, International Innovation Center for Forest Chemicals and Materials, College of Materials Science and Engineering, Nanjing Forestry University, Nanjing 210037, China; 3 School of Materials and Chemical Engineering, Ningbo University of Technology, Ningbo 315211, China

**Keywords:** sandwich structure, electromagnetic interference (EMI) shielding, silver nanowires (AgNWs), MXene

## Abstract

A thin, lightweight and flexible electromagnetic interference (EMI) shielding paper composite is an urgent need for modern military confrontations. Herein, a sandwich-structured EMI shielding paper composite with an easy pavement consisting of a filter paper layer, middle AgNWs/MXene layer, and polyvinyl butyral (PVB) layer was constructed by vacuum-assisted filtration, spraying and air-drying. The middle AgNWs/MXene compound endowed the filter paper with excellent electrical conductivity (166 S cm^−1^) and the fabricated filter paper–AgNWs/MXene–PVB composite exhibits superior EMI shielding (30 dB) with a 141 μm thickness. Remarkably, the specific EMI shielding effectiveness (SSE/t) of the filter paper–AgNWs/MXene–PVB composite reached 13,000 dB cm^2^ g^−1^ within the X-band frequency range. This value represents one of the highest reported for cellulose-based EMI shielding materials. Therefore, our sandwich-structured filter paper composite with superior EMI shielding performance can be used in the medical and military fields.

## 1. Introduction

With the advancement of contemporary society, daily life is increasingly dominated by a plethora of electronic devices, which, while facilitating routine activities, also contribute to a significant amount of electromagnetic pollution. Many studies have indicated that long-time exposure to electromagnetic waves may adversely affect human health. Therefore, investigations of electromagnetic interference (EMI) shielding materials have emerged as a prominent area of research in recent years [1,2,3,4,5,6].

Recently, biomass materials have been used to fabricate EMI shielding materials using a top-down method or bottom-up strategy [7]. Wood, characterized as a sustainable biomass resource, stands out as an excellent prospect for electromagnetic interference (EMI) shielding materials due to its cost-effectiveness, lightweight nature, and inherently porous and stratified structural properties [8,9,10,11]. A diverse array of carbon composites derived from wood have been engineered to yield high-performance EMI shielding materials, demonstrating considerable shielding effectiveness [12,13]. However, the above-fabricated materials are bulky and hard to use in practical applications. A bottom-up strategy can transform wood-derived biomass materials (mainly cellulose and lignin) into flexible and mechanically strong films/papers with superior EMI shielding properties [14]. However, this fabrication process is time-consuming, toxic, expensive, and hard to adapt to large-scale production. Another example is cellulose-based carbon aerogel [15,16,17]. Although these aerogels are lightweight, insulating and have good EMI shielding properties, their construction also comes with the aforementioned drawbacks. Moreover, these aerogels are commonly brittle and demonstrate poor physical and mechanical performance.

The paper making industry has a well-established history of using cellulose materials, including wood and recycled paper, to fabricate new paper via a sequence of processing and manufacturing steps. With the arrival of the Industrial Revolution, the paper industry grew to a larger scale and implemented greater efficiency. Traditional papermaking methods were gradually replaced by mechanization and automation, resulting in significantly increased production capacity. Currently, a multitude of paper products find utility across diverse sectors, encompassing decoration, automotives, and aerospace, among others. Therefore, the direct employment of paper substrates for producing EMI shielding materials will be practical and favorable. More recently, MXene and silver nanowires (AgNWs) have proved particularly suitable for EMI shielding applications, owing to their remarkable electrical conductivity and solution compatibility [16,18,19,20]. They can be coated on the surface of the matrix to fabricate superior EMI shielding materials. Unfortunately, the coating can easily be destroyed by physical friction, causing a low EMI SE. Polyvinyl butyral (PVB) is a prevalent thermoplastic resin frequently used to improve mechanical properties, thermal stability, and water- and oil-proofing performance [21].

In this study, we present an innovative and promising technique for fabricating large-format electromagnetic interference (EMI) shielding paper composites utilizing a vacuum impregnation, spray deposition, and evaporation drying strategy. Readily available filter paper serves as the substrate, with MXene/AgNWs acting as the intermediate functional layer, and an external layer of polyvinyl butyral (PVB) providing protection. This layered configuration bestows the filter paper–AgNWs/MXene–PVB composite with superior EMI shielding effectiveness (SE) along with robust physical and mechanical characteristics.

## 2. Materials and Methods

### 2.1. Materials and Chemicals

The filter paper (diameter: 60 mm; pore size: 20~25 μm) and hydrophilic PTFE microporous membrane were purchased from Cytiva (Shanghai, China). Sulfuric acid (H_2_SO_4_, 72%), ethanol (99.7%), acetone (99.5%), iron chloride (FeCl_3_, 99.9%), silver nitrate (AgNO_3_), ethylene glycol (EG), polyvinyl pyrrolidone (PVP), and polyvinyl butyral (PVB) were purchased from Aladdin (Shanghai, China). A delaminated solution of titanium carbide (Ti_3_C_2_ MXene solution, 5 mg/mL) was purchased from Jilin 11 Technology Co., Ltd. (Changchun, China).

### 2.2. Synthesis of Silver Nanowires (AgNWs)

AgNWs were prepared using a modified polyol method based on previous reports [12]. Specifically, 0.2 g PVP was absolutely dissolved in 25 mL EG under magnetic stirring at room temperature. Then, 0.25 g AgNO_3_ was added into the PVP/EG solution and stirred to form a transparent, uniform solution. Afterward, 3.5 g 0.6 mmol/L FeCl_3_ salt solution was dripped into the above mixture with stirring for 10 min to produce a uniform solution. Finally, the mixed solution was transferred into oil bath reactor (180 °C) for 45 min to grow AgNWs at a slow stirring speed. After the end of reaction, generated AgNWs were purified five times using a solvent exchange method with acetone and ethanol with the aid of centrifugation (5000 rpm, 5 min for each time and type of instrument). Finally, the AgNW precipitate was redispersed in ethanol with a concentration of 2.35 mg/mL for use. The microstructure of the AgNWs resembles long rods (Figure S1).

### 2.3. Preparation of Sandwich-Structured Filter Paper–AgNWs/MXene–PVB Composite

MXene solution was homogeneously dispersed in deionized water (5 mg/mL) and AgNWs was dispersed in ethanol (2.35 mg/mL) for after use. The filter paper was placed on the PTFE microporous membrane (0.22 μm pore size), and then 1.82 mL MXene solution and 0.39 mL AgNWs solution were applied and filtered through vacuum-assisted filtration, resulting in the formation of an MXene/AgNWs composite film. After that, the filter paper–MXene/AgNWs was peeled off from the filter, followed by air-drying. In the final stage, the air-dried filter paper–MXene/AgNWs composite film underwent a spraying process with 2 mL of a 2 wt% PVB solution, culminating in the creation of a functional filter paper–MXene/AgNWs–PVB (FMAP) composite for EMI shielding applications. The density of the fabricated FMAP is 0.73 g cm^−3^.

### 2.4. Characterizations

The lignin contents (Klason lignin) of the filter paper were determined by following a standard TAPPI T 222 om^−2^ method [22]. The Fourier transform infrared (FTIR) spectra of filter paper, filter paper–MXene/AgNWs, and filter paper–MXene/AgNWs–PVB were obtained using a Fourier transform infrared spectrometer (VERTEX 80 V, Bruker, Bremen, Germany) from 4000 to 400 cm^−1^ at a spectral resolution of 6 cm^−1^ and a total of 32 scans. The morphologies and microstructures of AgNWs, filter paper, filter paper–MXene/AgNWs and filter paper–MXene/AgNWs–PVB were observed by Phenom scanning electron microscopy (SEM) (Phenom XL G2, Phenom-World BV, Eindhoven, The Netherlands). A Four-Point-Probe instrument (Guangzhou Four-Point-Probe Technology, SDY-4, Guangzhou, China) was used to test the conductivity of samples. At least four parts were tested for all samples, and the average and standard deviation were reported. A Vector Network Analyzer (Agilent Technologies N5063A, Palo Alto, CA, USA) was used to measure the EMI shielding effectiveness in the frequency range of 8.2–12.4 GHz (X-band) (Figure S2), according to our previous study [23].

### 2.5. EMI Shielding Parameters

According to Schelkunoff theory, the total EMI shielding effectiveness (SE_T_) consists of absorption (SE_A_), reflection (SE_R_), and multi-reflection (SE_M_), where SE_M_ is often ignored when the SE_T_ is over 15 dB. The S-parameter is derived from the wave quantities a and b of the incident and reflected waves in the vector analysis tester.

(1) $$S_{11}=\frac{b_{1}}{a_{1}}|a_{2}=0$$

(2) $$S_{21}=\frac{b_{2}}{a_{1}}|a_{2}=0$$

(3) $$S_{12}=\frac{b_{1}}{a_{2}}|a_{1}=0$$

(4) $$S_{11}=\frac{b_{1}}{a_{2}}|a_{1}=0$$

The reflection coefficient R, the absorption coefficient A, and the transmission coefficient T are calculated from the S-parameters S11 and S21.

(5) $$R={\left|S_{11}\right|}^{2}$$

(6) $$T={\left|S_{21}\right|}^{2}$$

(7) A=1−T−R

Reflection is the main mechanism of shielding, which occurs at the interface of two different media with different refractive index or impedance characteristics. The reflection loss is given by the Frensel equation, as shown in the following equation:

(8) $${SE}_{R}=-10lg\left(1-R\right)=-10lg\left(1-{\left|S_{11}\right|}^{2}\right)$$

Absorption attenuation is given by the following equation:

(9) $${SE}_{A}=-10lg\left(\frac{T}{\left(1-R\right)}\right)=-10lg\left(\frac{\left|{\left|S_{21}\right|}^{2}\right|}{1-{\left|S_{11}\right|}^{2}}\right)$$

## 3. Results

**Figure 1:**
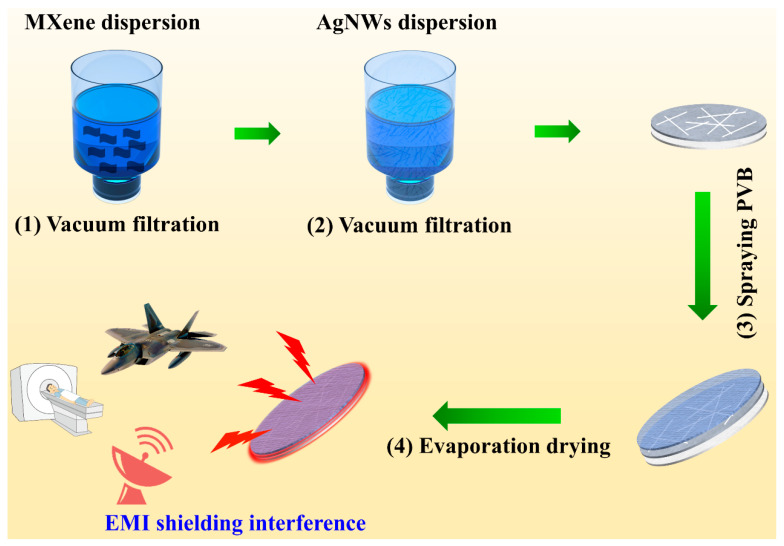
Schematic illustration for fabrication of the sandwich-structured filter paper–AgNWs/MXene–PVB composite.

**Figure 2:**
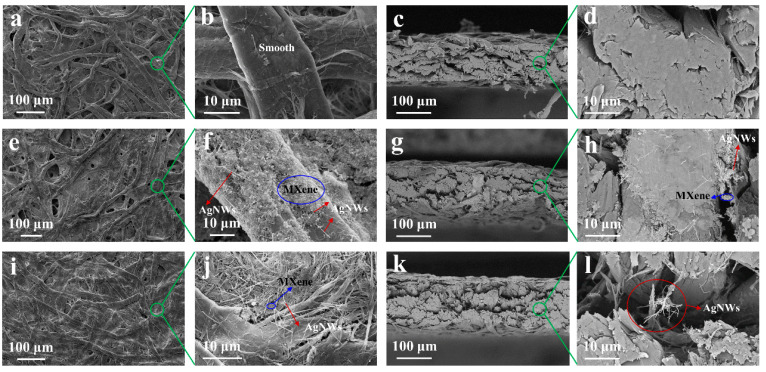
(**a**) Surface microstructure of filter paper and (**b**) its magnified SEM image. (**c**) Cutting surface morphologies of filter paper and (**d**) corresponding magnified SEM image. (**e**) Surface microstructure of filter paper–MXene/AgNWs sample and (**f**) its magnified SEM image. (**g**) Cutting surface morphologies of filter paper–MXene/AgNWs sample and (**h**) corresponding magnified SEM image. (**i**) Surface microstructure of filter paper–MXene/AgNWs–PVB sample and (**j**) its magnified SEM image. (**k**) Cutting surface morphologies of filter paper–MXene/AgNWs–PVB sample and (**l**) corresponding magnified SEM image.

The schematic for fabrication of the sandwich structural filter paper–AgNWs/MXene–PVB composite for electromagnetic shielding is depicted in Figure 1. Specifically, the filter paper was positioned beneath the Buchner flask, and then MXene solution and AgNWs solution were vacuum-filtered to fabricate the filter paper–MXene/AgNWs composite (FMA). Next, the FMA was peeled off from the filter flask followed by spraying with PVB and evaporation–drying to obtain the filter paper–MXene/AgNWs–PVB composite (FMAP). In this work, SEM was applied to investigate the surfaces of materials [24,25,26,27,28,29]. Figure 2 shows the morphologies of the filter paper, FMA, and FMAP. Figure 2a–d shows the microstructure of the pure filter paper. The filter paper was fabricated using smooth fibers through the papermaking process. After vacuum filtration of the MXene/AgNWs solution, the top surface of the filter paper was covered by the MXene layer and AgNWs rods. More importantly, the MXene and AgNWs were inserted into the inner of the filter paper through the cutting surface morphologies of FMA (Figure 2e–h). After PVB coating, the filter paper became denser, and we also observed that the PVB formed a membrane-like layer on the top surface of FMA. Meanwhile, the MXene and AgNWs were not influenced by the PVB membrane, having a good intrinsic morphology, which plays an important role in EMI SE.

**Figure 3:**
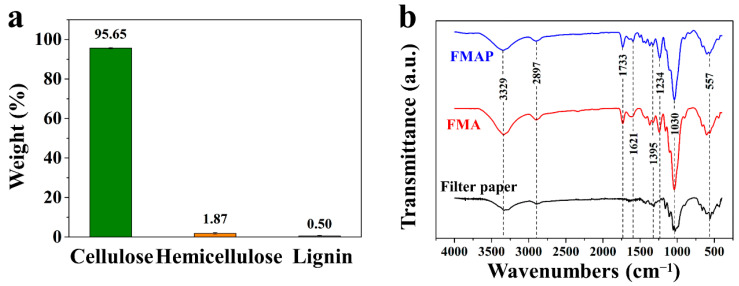
(**a**) Chemical composition of filter paper. (**b**) FTIR spectra of filter paper, FMA, and FMAP samples.

The chemical composition of the filter paper was analyzed using the standard Technical Association of the Pulp and Paper Industry (TAPPI)T 222 om^−2^ method. The result showed the filter paper is composed of 95.65% cellulose, 1.87% hemicellulose, and 0.50% lignin (Figure 3a), suggesting that the cellulose is the most dominated by matrix, which facilitates its mechanical flexibility. FTIR was used to obtain an infrared spectrum of the absorption or emission of the solid, liquid, or gas, which is helpful in determining the chemical structures of materials [30,31,32,33,34,35,36]. The FTIR spectra of the filter paper, FMA sample, and FMAP sample are displayed and compared in Figure 3b. Obvious characteristic peaks appear at 3329 cm^−1^ (O–H stretching vibration), 2897 cm^−1^ (C–H stretching vibration), and 1030 cm^−1^ (C–O stretching vibration) of cellulose, respectively. In addition, there are small peaks at 1733 cm^−1^, which correspond to the unconjugated carbonyl C=O of hemicellulose. The above analysis shows that the filter paper is mainly composed of cellulose, which is in accordance with the result of composition data. Commonly, silver itself does not contain organic elements, leading to no obvious absorption peak in the FTIR spectra. Yet, the surface of MXene has several characteristic peaks at 1395 cm^−1^, 557 cm^−1^, 1621 cm^−1^, 1234 cm^−1^, and 1030 cm^−1^ belonging to the C–F, –OH, C=O of alkone, C=C–O, and C–O–C stretching vibration functional groups. These typical peaks actually appeared in the FTIR spectra of FMA sample and FMAP sample, which indicated that there is a good connection between the filter paper and the AgNWs/MXene composite.

**Figure 4:**
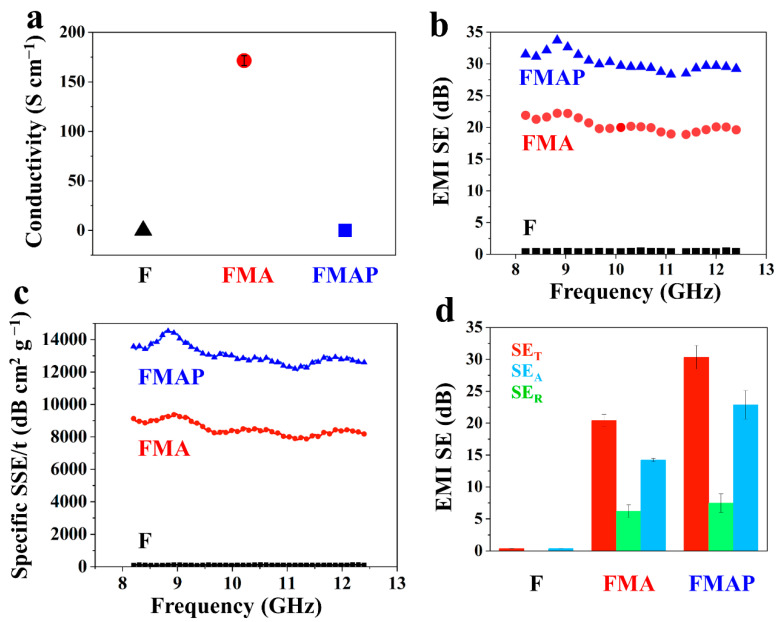
(**a**) Electrical conductivities of the F (filter paper), FMA, and FMAP samples. (**b**) EMI SE_T_ and (**c**) the corresponding specific SST/t of the F, FMA and FMAP samples. (**d**) Comparison of the SE_T_, SE_A_, and SE_R_ of the aforementioned specimens.

Conductivity is a critical factor influencing the electromagnetic interference (EMI) shielding performance of materials. The unmodified filter paper is dielectric (=0 S cm^−1^) (Figure 4a), having no EMI shielding effectiveness (~0 dB) (Figure 4b). After depositing conductive MXene and AgNWs on the surface of filter paper, the fabricated FMA sample shows superior conductivity with 166 S cm^−1^ (Figure 4a), further exhibiting good EMI shielding effectiveness. The total shielding effectiveness (SE_T_) of FMA achieves 21 dB at a thickness of 141 μm in the 8.2–12.4 GHz frequency range (Figure 4b). To provide a more comprehensive assessment of material performance, the specific EMI shielding effectiveness (SSE/t), calculated by dividing SE by density and thickness, is utilized. As depicted in Figure 4c, the EMI SSE/t values of FMA sample are above 8000 dB cm^2^ g^−1^. It is well known that the surface coatings of MXene and AgNWs are susceptible to damage, potentially leading to a loss of EMI shielding effectiveness. To address this issue, the FMA sample was coated with a layer of polyvinyl butyral (PVB), known for its high physical and mechanical strength, thus forming the FMAP composite MXene. Then, the conductivity and EMI shielding effectiveness of FMAP were evaluated, and the results showed that the FMA was covered by dielectric PVB (the conductivity of FMAP = 0 S cm^−1^) (Figure 4a). Surprisingly, the EMI shielding effectiveness did not decrease; conversely, it increased to up to 30 dB (Figure 4b), and the specific EMI SE of FMAP reached a very high value of 13,000 dB cm^2^ g^−1^ (Figure 4c). In addition, there was greater absorption efficiency (SE_A_) than reflection efficiency (SE_R_) in the FMA and FMAP samples, indicating SE_A_ plays a more important role than SE_R_ in EMI SE. The result also shows that MXene/AgNWs are more effective in terms of EMI SE through enhanced SE_A_ due to the PVB protection effect (Figure 4d). The reason is that when PVB is sprayed on paper, ethanol will loosen AgNWs and Mxene at the interface, and after drying again, more bonding points will appear at the interface, which is beneficial for improving absorption efficiency (SE_A_). In order to make a good comparison, we also tested the EMI SE of the carbon pencil and explained our reasoning (Figure S3).

## 4. Conclusions

In summary, this work showcases the fabrication of a sandwich-structured filter paper–MXene/AgNWs–PVB composite for high-performance electromagnetic interference shielding. This was achieved through a two-step vacuum-assisted filtration process, followed by a methodical spraying and evaporation drying technique. An exhaustive examination of the filter paper–MXene/AgNWs–PVB composite’s chemical structure, microstructure, electrical conductivity, and EMI shielding effectiveness was undertaken. The middle MXene/AgNWs layer showed a high electrical conductivity of 166 S cm^−1^, achieving good EMI SE (30 dB) and a high SSE/t value (13,000 dB cm^2^ g^−1^) in the filter paper–MXene/AgNWs–PVB composite. The composite’s comprehensive properties make it an ideal candidate for future applications in smart homes and the aerospace, military, and artificial intelligence domains.

## Data Availability

The data presented in this study are available upon request from the corresponding author. The data are not publicly available due to privacy restrictions.

## Supplementary Materials

The following supporting information can be downloaded at: https://www.mdpi.com/article/10.3390/polym16060760/s1, Figure S1: (1) SEM image of AgNWs; (2) TEM micrograph of MXene nanosheets. Figure S2: Vector Network Analyzer (Agilent Technologies N5063A, Palo Alto, State of California, USA) was used to measure the EMI shielding effectiveness in the frequency range of 8.2−12.4 GHz (X-band). The cross-section size is 22.86 mm × 10.16 mm. Figure S3: Comparison of SET, SEA, and SER of the F, FMA, FMAP and FC samples.
