# Supplementary material for: A Sandwich Structural Filter Paper–AgNWs/MXene Composite for Superior Electromagnetic Interference Shielding
Source: Polymers (Basel). 2024 Mar 10;16(6):760. doi: 10.3390/polym16060760 (PMC10975682; doi:10.3390/polym16060760)
Supplement: Supplementary file 1 [file polymers-16-00760-s001.zip › polymers-2745369-supplementary.pdf]

A Sandwich Structural Filter Paper–AgNWs/MXene Composite for Superior Electromagnetic Interference Shielding

Xiaoshuai Han [1,2], Hongyu Feng [2], Wei Tian [2], Kai Zhang [1], Lei Zhang [1], Jiangbo Wang [3] and Shaohua Jiang [2,*]

1 State Key Laboratory of Biobased Material and Green Papermaking, Qilu University of Technology, Shandong Academy of Sciences, Jinan 250353, China; xiaoshuai.han@njfu.edu.cn (X.H.)
2 Jiangsu Co-Innovation Center of Efficient Processing and Utilization of Forest Resources, International Innovation Center for Forest Chemicals and Materials, College of Materials Science and Engineering, Nanjing Forestry University, Nanjing 210037, China
3 School of Materials and Chemical Engineering, Ningbo University of Technology, Ningbo 315211, China
* Correspondence: shaohua.jiang@njfu.edu.cn; Tel.: +86-156-2496-0675

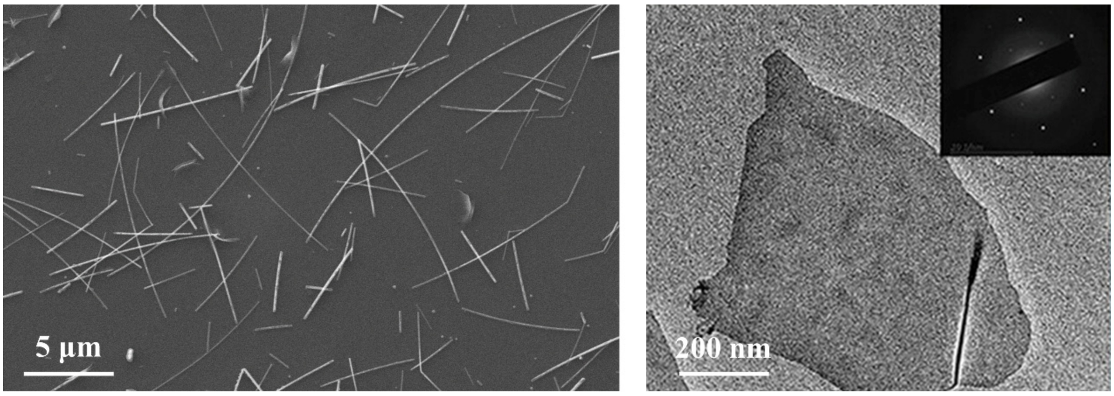

Figure S1 (1) SEM image of AgNWs; (2) TEM micrograph of MXene nanosheets.

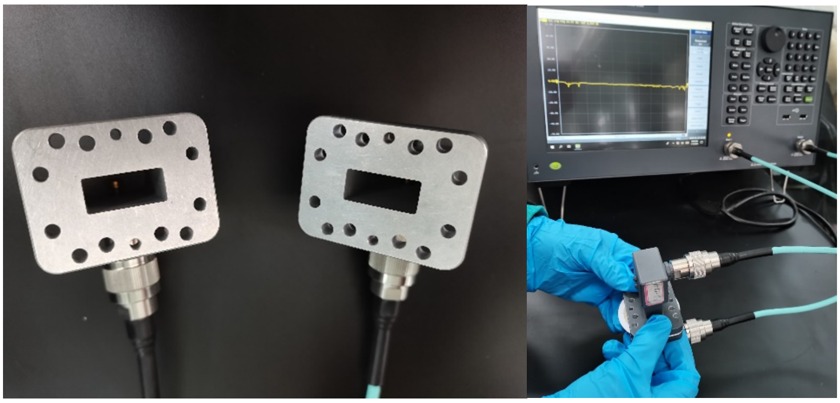

Figure S2 Vector Network Analyzer (Agilent Technologies N5063A, Palo Alto, State of California, USA) was used to measure the EMI shielding effectiveness in the frequency range of 8.2−12.4 GHz (X-band). The cross-section size is 22.86 mm × 10.16 mm.

The experimental data were measured by the E5063A ENA vector network analyzer, which has a scanning speed of up to 201 points for 9 ms, a maximum frequency of 18 GHz, a dynamic range of 117 dB, a number of built-in ports of 2 ports, and an output power and trace noise of 0 dBm and 0.015 dB rms, respectively.

When we use the Vector Network Analyzer E5063A to measure the membrane-related properties, the waveguide unit excites TE (Transverse Electric) mode waves. When the TE mode wave is transmitted in the rectangular waveguide, the electric field is perpendicular to the cross-section direction of the waveguide, and the magnetic field is mainly located in the cross-section direction. Its propagation direction in the waveguide is perpendicular to the propagation direction of the waveguide. The electric field component of the wave in this mode does not penetrate the waveguide wall during transmission, making it relatively suitable for the measurement of electromagnetic shielding of membranes. Therefore, when E5063 A is connected to a rectangular waveguide to measure the electromagnetic shielding of the film, the waveguide unit will excite the TE wave, and then the vector analyzer is used to measure the absorption, reflection, transmission and other characteristics of the wave propagating in the sample, so as to evaluate the shielding effect of the film on the electromagnetic wave.

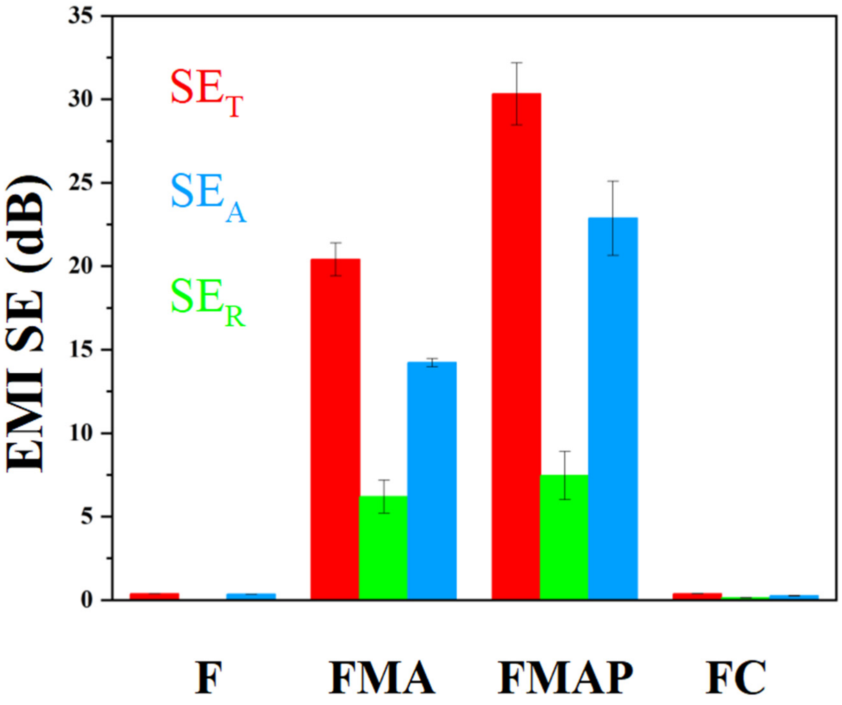

Figure S3. Comparison of SET, SEA, and SER of the F, FMA , FMAP and FC samples.

Coating filter paper with Carbon Pencil enhances its electromagnetic shielding properties, but has limited effect. Carbon pencils contain carbon, which has good electrical conductivity and can form a conductive film on the surface. Such a film can absorb or reflect electromagnetic radiation to a certain extent, thereby attenuating the penetration of electromagnetic waves. Therefore, applying the carbon pencil to the filter paper can increase the conductivity of the filter paper and improve its shielding ability against electromagnetic radiation. However, it should be noted that the shielding effect of the carbon pencil coated on the filter paper is relatively weak, especially for higher frequency electromagnetic radiation. In addition, factors such as the uniformity and thickness of the coating, as well as the quality of the carbon pencils, can also affect the shielding performance. For more demanding electromagnetic shielding applications, more specialized shielding materials or structures may be required. The filter paper coated with carbon pencil is marked as FC.
